# Assessing Feedback Response With a Wearable Electroencephalography System

**DOI:** 10.3389/fnhum.2019.00258

**Published:** 2019-07-25

**Authors:** Jenny M. Qiu, Michael A. Casey, Solomon G. Diamond

**Affiliations:** ^1^Thayer School of Engineering, Dartmouth College, Hanover, NH, United States; ^2^Department of Music, Dartmouth College, Hanover, NH, United States

**Keywords:** applied neuroscience, EEG, P3, N2, FRN, brain computer interfaces

## Abstract

**Background:** Event related potential (ERP) components, such as P3, N2, and FRN, are potential metrics for assessing feedback response as a form of performance monitoring. Most research studies investigate these ERP components using clinical or research-grade electroencephalography (EEG) systems. Wearable EEGs, which are an affordable alternative, have the potential to assess feedback response using ERPs but have not been sufficiently evaluated. Feedback-related ERPs also have not been scientifically evaluated in interactive settings that are similar to daily computer use. In this study, a consumer-grade wearable EEG system was assessed for its feasibility to collect feedback-related ERPs through an interactive software module that provided an environment in which users were permitted to navigate freely within the program to make decisions.

**Methods:** The recording hardware, which costs < $1,500 in total, incorporated the OpenBCI Cyton Board with Daisy chain, a consumer-grade EEG system that costs $949 USD. Seventeen participants interacted with an oddball paradigm and an interactive module designed to elicit feedback-related ERPs. The features of interests for the oddball paradigm were the P3 and N2 components. The features of interests for the interactive module were the P3, N2, and FRN components elicited in response to positive, neutral, and two types of negative feedback. The FRN was calculated by subtracting the positive feedback response from the negative feedback responses.

**Results:** The P3 and N2 components of the oddball paradigm indicated statistically significant differences between infrequent targets and frequent targets which is in line with current literature. The P3 and N2 components elicited in the interactive module indicated statistically significant differences between positive, neutral, and negative feedback responses. There were no significant differences between the FRN types and significant interactions with channel group and FRN type.

**Conclusion:** The OpenBCI Cyton, after some modifications, shows potential for eliciting and assessing P3, N2, and FRN components, which are important indicators for performance monitoring, in an interactive setting.

## 1. Introduction

The development of smaller and power efficient electronics over the last few decades has facilitated the growth of lower cost, space-efficient, wearable versions of common medical devices. With the market for wearable consumer products currently valued at $12.3 billion and projected to increase to $30.7 billion by 2021, consumers will increasingly have access to different health metrics, such as cardiac activity and caloric output, within reach. The consumer demand for better wearable devices and more interesting metrics subsequently drove the development of low-cost, wearable neural systems that use electroencephalography (EEG), a non-invasive, highly temporal, imaging technique that monitors electrical activity of the brain through electrodes that are placed on the scalp (Mak and Wolpaw, 2009; van Gerven et al., 2009; McFarland and Wolpaw, 2011; Nicolas-Alonso and Gomez-Gil, 2012; Daly and Huggins, 2015; Lee, 2016). As wearable, low-cost EEG systems become more prevalent and available to consumers, there is a need to assess the potential applications and capabilities of these low-cost EEG systems in measuring neural activity. The present article benchmarks one of these low-cost EEG systems, the OpenBCI V-32 Cyton with Daisy Chain (OpenBCI, 2018), in a visual oddball task and in an interactive, feedback-related event-related potential (ERP) study. The translational extensions of this work are to deploy consumer-accessible, neural-based performance monitoring and expand the environments in which feedback-related ERP components are monitored.

Wearable EEG systems have distinct advantages over clinical EEG systems. Traditional clinical-grade EEG systems are expensive, with the average system costing over $50k for hardware and $20k for processing software (Brain Vision, 2017). The application time for traditional EEG electrodes can exceed an hour from assembly and experimental set-up. In comparison, consumer-grade EEG systems typically range from $500 to $1,000 for hardware depending on available features with an additional $50 fee for software (McFarland and Wolpaw, 2011; Shih et al., 2012; Badcock et al., 2013; Ratti et al., 2017). With user friendly headsets and fewer electrodes, low-cost EEG systems can have a short set-up time of 20 min or less (Krigolson et al., 2017; Ratti et al., 2017). In studies comparing various wireless systems, such as Emotiv, Muse, and openBCI to clinical systems, such as Biosemi and ActiChamp, wireless systems were shown with a significantly lower setup time of 5–6 min while traditional clinical-grade systems took 15–20 min for setup (Vourvopoulos and Badia, 2016; Krigolson et al., 2017).

Several studies have demonstrated that low-cost EEG systems are capable of collecting neural signals comparable to the quality of those collected from clinical EEG systems (Debener et al., 2012; Badcock et al., 2013; De Vos et al., 2014; Frey, 2016; Krigolson et al., 2017; Ratti et al., 2017; Dehais et al., 2019). In such studies, these low-cost EEG systems are often benchmarked in classic experiments that elicit event related potentials (ERP), which are time-locked series of peaks resulting from an averaged electrical response from large groups of neurons (Robertson and Pascual-Leone, 2003; Schendan et al., 2003; Cassidy, 2004; Maia, 2009; Bassett et al., 2011; Walsh and Anderson, 2012; Schuck et al., 2017). The most common benchmark, the oddball paradigm, elicits an ERP with a distinctly large positive peak, known as the P300 or P3, located 300–500 ms after the presentation of an infrequent, target stimulus (Squires et al., 1975). In literature involving oddball paradigms as measured by clinical EEG systems, the P3 amplitude is influenced positively by lower probability, and distinctiveness of the stimuli (Linden, 2005; Graimann et al., 2010; Luck, 2014). The N200 or N2, a negative inflection occurring 200–350 ms after stimulus presentation, has been less investigated with respect to the oddball paradigm. The N2 amplitude has been determined to be larger for task relevant stimuli and for lower probabilities of task relevant stimuli (Squires et al., 1975; Warren et al., 2011; Weschke and Niedeggen, 2016). Researchers have demonstrated that the ERPs elicited in both auditory oddball paradigms (Debener et al., 2012; Badcock et al., 2013) and visual oddball paradigms (Frey, 2016; Krigolson et al., 2017), have been highly correlated to each other (Badcock et al., 2013; De Vos et al., 2014; Frey, 2016; Vourvopoulos and Badia, 2016; Ratti et al., 2017). The combined advantages of the wireless EEG system have allowed researchers to expand the realm of neural monitoring studies to outside standard clinical testing areas. Researchers have used wireless consumer-grade EEG systems in conjunction with the oddball paradigm in walking experiments (Debener et al., 2012), flight training in planes (Callan et al., 2018; Dehais et al., 2019), and in extended monitoring situations (Debener et al., 2015) demonstrating the potential for extended periods of neural monitoring in scenarios where the brain is actively at work. These specific studies also subsequently demonstrated that dry electrodes (Callan et al., 2018; Dehais et al., 2019) or adhesive minimal gel electrodes (Debener et al., 2015) are suitable alternatives to the traditional wet electrodes arrays.

However, wireless consumer-grade EEGs are not without significant drawbacks. Low-cost systems that are inflexible in electrode positioning have shown to be more prone to artifacts from muscle movements (Badcock et al., 2013; Ratti et al., 2017). A number of consumer-grade EEG systems that incorporate dry electrodes have a lower signal-to-noise ratios in comparison to wet or traditional electrode systems (De Vos et al., 2014; Mihajlovic et al., 2015; Lin et al., 2016; Zerafa et al., 2018; Kam et al., 2019). Wireless, consumer-grade EEG systems can be delayed in comparison to wired, traditional EEG systems. In one study that compared the clinical-grade g.tec g.USBamp to the OpenBCI in classification capability, the OpenBCI had a statistically significant difference in the area under the receiving operating characteristic curve (AUROCC) for time-sensitive ERP tasks. However, the significant difference was speculated to be partially explained by a software issue that created a time delay of 88 ms for the OpenBCI (Frey, 2016).

Considering both the advantages and disadvantages, the wireless consumer-grade EEGs should be capable of measuring other time-related neural signals, such as feedback related ERPs. User feedback gives individuals the opportunity to modify their future actions using information received about their previous behaviors. The most studied ERP component related to feedback response is feedback-related negativity (FRN) (Walsh and Anderson, 2012; Warren and Holroyd, 2012; Gheza et al., 2018; Glazer et al., 2018; Krigolson, 2018; Somon et al., 2019). The FRN is a signal that is said to originate in the anterior cingulate cortex (ACC) by multiple converging studies (Walsh and Anderson, 2012; Warren and Holroyd, 2012; Gheza et al., 2018; Glazer et al., 2018). The FRN is associated most strongly with the N2 component. The FRN has been represented as a comparison between measured N2 peaks on ERPs elicited after feedback (Sailer et al., 2010) and, more commonly, as a difference waveform between averaged ERPs formed from negative feedback and averaged ERPs formed from positive feedback. The amplitude of the N2 component, and subsequently the FRN component, can be affected by prevalence of the stimulus, perceived potential gain or loss, and conflict of information (Gheza et al., 2018; Glazer et al., 2018; Krigolson, 2018). The N2 is generally larger in an ERP response after negative feedback than for that of positive feedback (Walsh and Anderson, 2012; Warren and Holroyd, 2012; Gheza et al., 2018; Glazer et al., 2018; Krigolson, 2018; Somon et al., 2019). The amplitude of the P300 (P3) component has also been studied in performance monitoring research. Slightly larger P3 peaks were sometimes attributed to more positive feedback but were generally not statistically significant (Potts, 2011; Yi et al., 2018; Schindler et al., 2019; Tunison et al., 2019). One study compared a consumer-grade MUSE EEG system and the clinical-grade Brain Vision ActiChamp system and found that the FRN responses were similar in a selection task (Krigolson et al., 2017).

The purpose of the present study is to demonstrate that a consumer-grade EEG system is appropriate for ERP research in feedback response in an interactive environment. In order to accomplish this, we first benchmark the EEG system with the standard oddball paradigm. Then, we test the EEG system in response to an interactive environment that is designed to elicit feedback related ERPs. We created an environment in which the user is free to navigate around a trial to solve a puzzle and make numerous decisions to learn about the solution. When a user makes a decision, the environment provides delayed feedback. We investigated the P3, N2, and the FRN components because these components are relatively large in comparison to other components. Our first objective for the study was to analyze P3 and N2 components from a low-cost wearable EEG system with respect to a standard oddball paradigm. Our expectation is that the low-cost wearable EEG would detect statistically significant larger N2 and P3 components from infrequent stimuli in a standard oddball paradigm, much like the results of clinical and wireless EEG systems. The second objective for the study was to analyze the N2, P3, and FRN components using the chosen low-cost wearable EEG system in an interactive environment. The second hypothesis was that the low-cost EEG system can detect statistically significant ERP components in reaction to feedback in an interactive module. When comparing ERP responses from negative and positive feedback, the negative feedback would elicit a larger N2 component and stronger negative feedback would elicit a larger FRN component than weaker negative feedback.

## 2. Methods

### 2.1. Participants

Twenty participants (nine females, two left-handed, age 22–33, *M* = 26.4) with less than two encounters with an EEG or EEG-based BCI provided informed consent to participate in this study. Three of these participants (three female, age 24–26, *M* = 25) were excluded from the analysis because of substantial noise from insufficient electrode connection to the scalp. All participants had normal or corrected vision, reported no prior incidents of neurological impairment, and were fluent in English. The study protocol was approved by the Dartmouth Institutional Review Board.

### 2.2. Materials

Each participant encountered two modules sequentially: (1) an oddball paradigm (OB) and (2) an interactive module (IM). Both modules lasted ~30 min and were programmed with Python and Psychopy packages (Peirce et al., 2019).

#### 2.2.1. Oddball Paradigm

In this visual oddball paradigm module, participants were presented with an image and subsequently identified using a mouse whether the image was an infrequent stimulus or the frequent stimulus. The materials for the oddball paradigm module were three unique infrequent stimuli that were chosen randomly from a library of 40 shapes colored with similar brightness and saturation and one frequent stimulus, which was a white cross. Each participant received a different set of infrequent stimuli and the same frequent stimulus in order to avoid potential confounds with stimulus-specific factors (Luck, 2014). All images were simple geometric shapes generated through python. The full list of chosen stimuli is included in the Supplementary Material. The module was created using Python and presented through Psychopy version 1.85.2.

#### 2.2.2. Interactive Module

**Figure 1:**
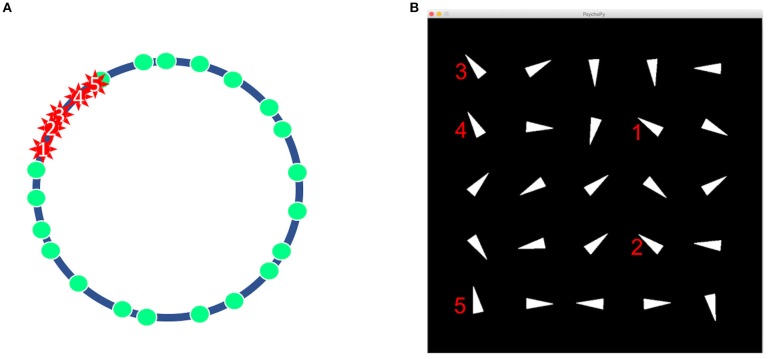
The design of a trial in the interactive module. **(A)** 20 non-mines and 5 mines represented as points on a circle. **(B)** Example of a trial as seen by participant. Notice how the five identified marks in **(A)** are displayed as triangles in an orientation in **(B)**.

The four stimuli selected for each participant from the oddball paradigm module were used as feedback representations to decisions made in the interactive module. The module was formulated to produce repeatable feedback-related ERPs in a less structured environment. To accomplish this, each trial of the module was designed as a puzzle. Each trial was represented as a set of 25 triangles that were pointing in different directions Figure 1B. The directions of each triangle corresponded to the value of 25 pseudo-random points that were relatively evenly distributed as shown in Figure 1A. Five points clustered together were randomly designated as mines. The objective for each trial was to determine which of the 25 triangles hid mines and which hid blank spaces. The program was coded through Python and presented through Psychopy version 1.85.2.

#### 2.2.3. Procedure

Experimental tasks were administered using Psychopy Version 1.85.2. Data collection occurred in a single session lasting 100–150 min. Each participant session began with a Mini Mental State Examination (MMSE) to test for mental fitness for the session (Folstein et al., 1975). After the electrode and experimental set up, each participant completed the oddball paradigm module. Through a standard set of instructions, each participant was directed to left click when the frequent stimulus appeared and to right click when the target infrequent stimulus appeared. After the participant indicated comprehension of the instructions, there was a fixation period in which a fixation cross was displayed in the center of the screen for 10 s. Each participant encountered 24 blocks of 20 trials yielding 480 trials in total. Within each block, one of the three infrequent stimuli that were assigned uniquely to the participant was presented to the participant as the “target stimulus.” For each trial, the stimulus was presented on a black background for 500 ms followed by 1,500 ms of black screen. Ten percent of the trials given to each participant contained one of the infrequent stimuli and the other 90% contained the frequent stimulus. The participant was given a rest period of 20 s after three blocks.

The interactive module was administered upon completion of the oddball paradigm. In the instructions, the participant was told that each trial consisted of 25 white triangles pointing in 25 different directions. The participant was asked to navigate within the trial to determine whether triangles hid mines or hid nothing. If the participant believed that the triangle of interest hid a mine, the participant would right click on the triangle. The participant would left click if the triangle was thought to hide a blank space. The module would then replace the triangle with a response stimulus after a delay. The three infrequent stimuli and the frequent stimuli used in a previous oddball paradigm module served as response stimuli indicating correctness or incorrectness with the right or left click. As the mines were clustered together as indicated in Figure 1A, the triangles hiding mines were orientated in the relatively similar directions. In order to successfully complete the 25 triangle problem with as few errors as possible, the participant needed to quickly distinguish the solution orientations by trial and error. The participant was awarded 10 points for correctly identifying a mine, 1 point for correctly identifying a blank space, −5 points for incorrectly identifying a blank space as a mine, and −10 points for incorrectly identifying a mine as a blank space. The participant was given the freedom to choose any triangle in any amount of time within the trial period. When the participant indicated a decision using the mouse, the feedback response stimulus was revealed after a waiting period of 1 s. At the end of clicking through all 25 triangles, the participant was given overall feedback on the round with the number of points accumulated and types of responses made. The participant encountered 30 trials with a total of 750 response stimuli from the module.

### 2.3. Data Acquisition and Processing

#### 2.3.1. Data Acquisition

Continuous EEG was recorded from 16 Ag-AgCl coated dry electrodes mounted on an in-house designed neoprene head cap. The channel positions used were FP1, FP2, F5, F1, F2, F6, FC3, FC4, C3, Cz, C4, P3, Pz, P4, O1, and O2. The system included a MacBook Pro Retina, 15-inch, Mid 2014 (Apple Inc), and an OpenBCI V3-32 Cyton Board, a low-cost EEG system (OpenBCI, 2018). There were two auxiliary inputs hard wired into the OpenBCI Cyton board from the mouse and from a photoresistor that was connected to the laptop screen. Data was streamed wirelessly at a sampling rate of 1,000 Hz using a PushTheWorld WiFi Shield and collected through Terminal using NodeJS. The recording electrodes were referenced online to the right mastoid with an ear clip electrode.

#### 2.3.2. EEG Modifications

**Figure 2:**
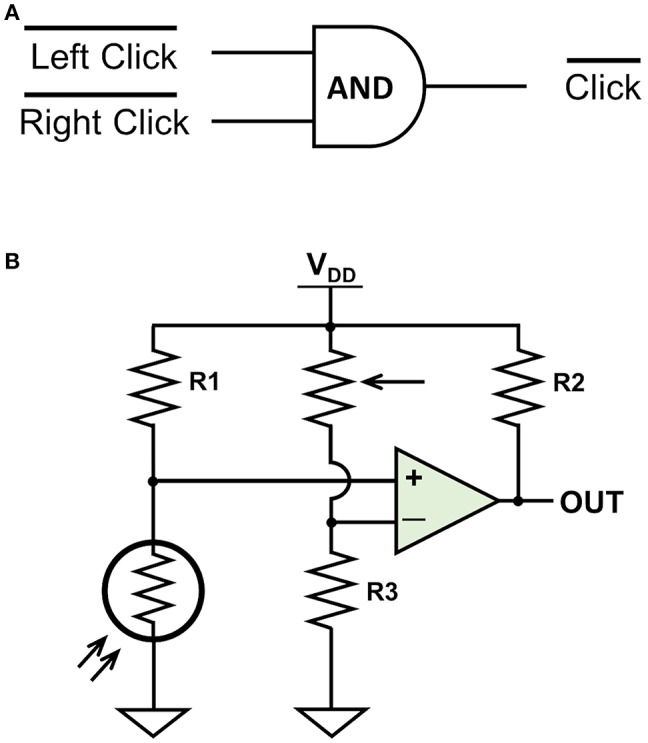
The primary circuits designed for auxiliary digital triggers. **(A)** The mouse circuit. **(B)** The photoresistor circuit. The mouse circuit sends a 1 when neither mouse button is clicked. When either the left click or right click is triggered, the corresponding side is connected to ground and a 0 is sent to the aux port. The photoresistor circuit sends a 1 when the photoresistor senses no light. The potentiometer and R3 form a resistive voltage divider to create an adjustable reference voltage that is fed into the negative side of the comparator. When the photoresistor detects light during stimulus onset, the voltage on the positive side of the comparator is higher than the reference voltage and a 0 is sent to the aux port.

The preloaded settings for the OpenBCI Cyton Board allowed three digital or two analog auxiliary inputs and marked each sample, which was transferred as a 32 byte EEG packet, with the time received by the computer. In order to remove the potential delay between sending and receiving, we activated a subroutine that was natively programmed into the OpenBCI board but not readily accessible. This subroutine provided the time for when each line of data was recorded on the board. This subroutine used four of the six designated auxiliary bytes which subsequently reduced the number of available digital auxiliary inputs from three to two. The first digital input was from a photoresistor connected to the laptop screen that recorded stimulus onset. The second digital input came from a rewired wireless mouse that routed circuits for the left and right mouse button through an AND gate. The circuits for both are shown in Figure 2. The photoresistor digital input recorded 1s when there were no events and 0 s when a stimulus occurred. The mouse digital input also recorded 1s when there were no events and 0s when a click occurred. Events were said to occur when the first 0 of each time period was recorded. The events as recorded by the EEG system were then matched with events as recorded by Psychopy.

The OpenBCI Cyton contains two rows of pins in which to insert recording, reference, and ground electrode connectors. The OpenBCI guide recommends using pins INxN, which are closest to the board, for all electrodes (OpenBCI Inc, 2019). According to the description sheet for the analog to digital converter placed on the board, these electrode pins are connected to the negative inputs (Texas Instruments, 2012). As a consequence, all recorded signals from electrodes using these pins have to be inverted prior to analysis.

### 2.4. Data Processing

Offline processing was conducted using a combination of Python and the EEGLAB v14 (Delorme and Makeig, 2004) and ERPLAB toolboxes (Mihajlovic et al., 2015) in MATLAB. All correct responses from the Oddball paradigm and all responses from the Interactive Module were processed for analysis. The continuous raw EEG data was first processed with a high-pass fourth order Butterworth filter at 1 Hz and subsequently processed with a low-pass fourth order Butterworth filter at 30 Hz. The data was then ported to MATLAB. Trials with excessive artifacts aside from eye blinks and eye movement were manually excluded. Independent component analysis (ICA) using BINICA from the EEGLAB v14 toolbox was used to isolate components related to blink artifacts. The removed components were identified through projected scalp topography, timing, and spectra (mean = 1.11). Trials were screened afterward to identify noisy electrodes (mean = 2.47) and to identify epochs with excessive drift. In total, 15% of correct trials for the oddball paradigm and 11.5% of the IM trials were excluded. The number of excluded trials did not differ significantly across conditions or subjects. The data was then inverted because of the connection scheme to the digital-to-analog converter as described in section 2.3.2. The power spectral density for both modules were calculated using the Welch's power spectral density estimate.

**Figure 3:**
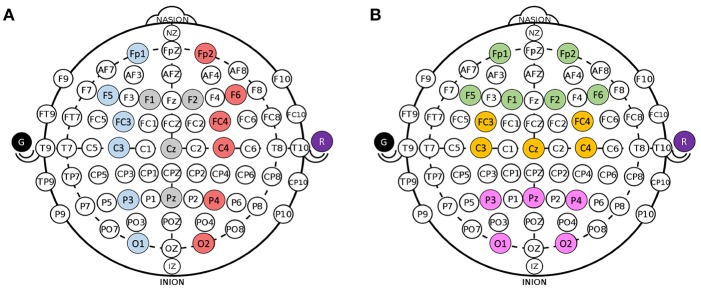
Groupings for electrodes. **(A)** Divisions for Left (blue), Center (gray), and Right (red) electrodes. **(B)** Divisions for Front (green), M (yellow), and Back (pink) electrodes. Ground (black) and Reference (purple) are collected from ear clip electrodes.

ERPs were calculated over a 1,500 ms window with a 500 ms pre-stimulus baseline and locked to the presented stimulus for all electrodes in all participants in both modules. The features of interest for the oddball paradigm were the P3 mean amplitude and the N2 mean amplitude. The P3 mean amplitude was measured between 300 and 700 ms and the N2 mean amplitude was measured between 200 and 300 ms (Luck, 2014). The feature of interest for the IM were also the P3 mean amplitude and the N2 mean amplitude as well as the FRN. ERPs were isolated within the modules and separated depending on the presented stimulus. As the consumer-grade EEG system compounded with dry electrodes have low signal to noise ratios, groups of electrodes were averaged together to increase power. All of the electrodes were divided into groups of four to six based on vertical regions [Left (L), Center (C), Right (R)] and horizontal regions [Front (F), Middle (M), Back (B)] and averaged together. The clusters are defined in Figure 3. In all statistical analyses, only parallel regions were compared.

All statistical analyses for EEG features were carried out using R (R Core Team, 2014). The mean amplitudes for both P3 and N2 means were analyzed through repeated measures ANOVA using the ez package (Lawrence, 2015). F-values were corrected based on Mauchly's Test for Sphericity and a Geiser-Greenhouse adjustment was performed if sphericity was violated. The statistical analysis was performed comparing only three electrode groups, either [L, C, R] or [F, M, B] at a time. The purpose of the statistical analysis for the oddball paradigm was to verify that the P3 response was elicited correctly for the infrequent stimuli and the frequent stimulus. Two repeated measures ANOVAs was performed with electrode region (3) and stimulus type (2) as factors for the oddball paradigm. To determine whether there were any significant differences between response type in the IM, two repeated measures ANOVAs were performed with electrode region (3) and stimulus type (4) as factors. The first followup analysis investigated differences between positive, negative, and neutral feedback in the IM using two repeated measures ANOVAs that were performed with electrode region (3) and feedback type (3) as factors. The second followup analysis investigated differences between the weak punishment and strong punishment FRN in the IM using two repeated measures ANOVAs with electrode region (3) and FRN type (2) as factors. Statistically significant results with more than two levels were followed by a *post-hoc* paired *t*-test with Bonferroni corrected *p*-values (Luck and Gaspelin, 2017). The behavioral data was also analyzed using the ez package in R. The predictors of interest was reaction time for the oddball paradigm and distribution of trial types for the interaction module.

## 3. Results

### 3.1. Oddball Paradigm

**Figure 4:**
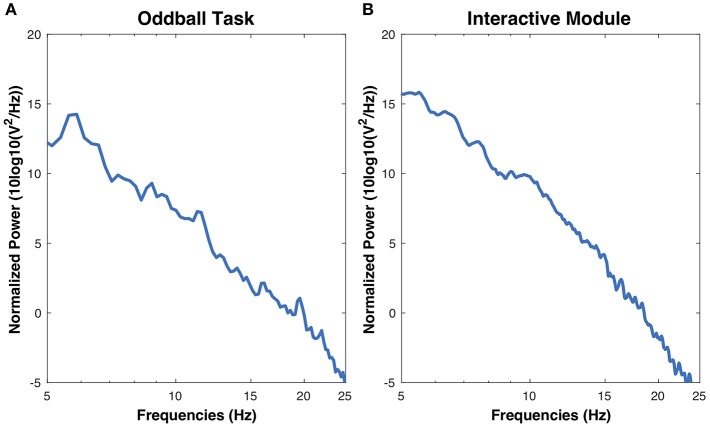
Power spectral densities estimations for the oddball task and the interactive module. PSDs for both were calculated using Welch's power spectral density estimation.

**Figure 5:**
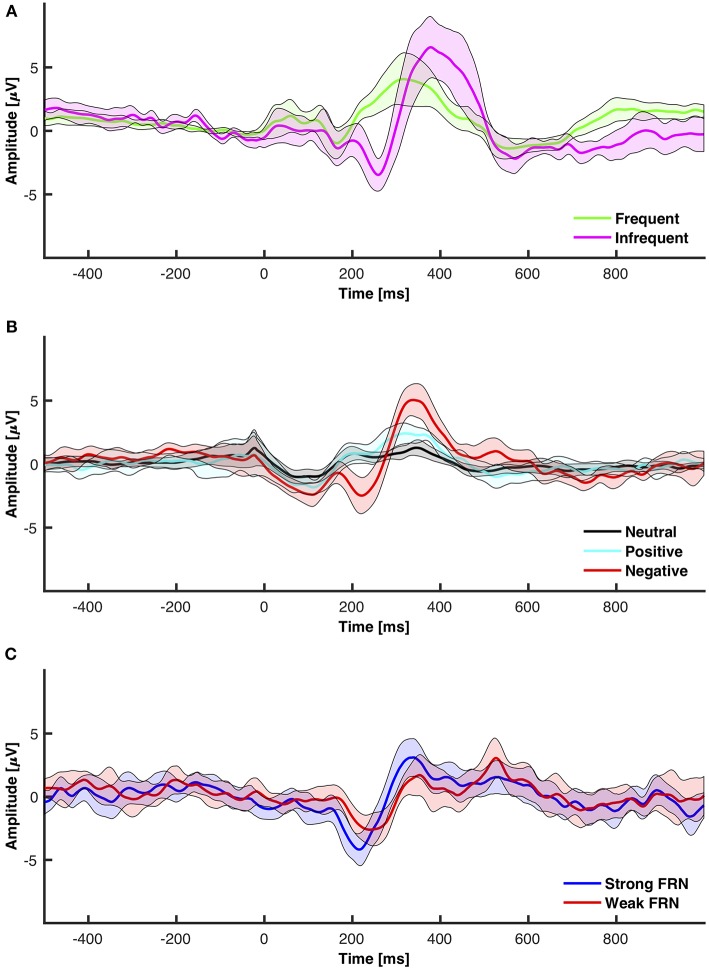
95% Confidence intervals for grand averaged event related potentials. **(A)** Frequent vs. infrequent ERP from the oddball paradigm. **(B)** Neutral (+1) vs. positive (+10) vs. negative (−5 and −10) from the interactive module. **(C)** FRN elicited from subtracting positive from negative.

The objective for this section was to analyze ERP components, specifically the P3 and the N2, from the chosen low-cost wearable EEG system in a standard oddball paradigm. ERP grand average waveforms are depicted in Figure 5A. The mean amplitudes and standard deviations for P3 and N2 are reported in Table 1. The power spectral density (PSD) is reported in Figure 4.

**Table 1:** The P3, N2, and FRN mean amplitudes from the oddball task and interactive module.

**Task**	**Test**	**Stimulus**	**Peak**	**Mean (μV)**	**SD (μV)**
Oddball	Infrequent vs. Frequent	Infrequent	P3	4.33	4.12
			N2	−2.63	2.15
		Frequent	P3	2.48	2.74
			N2	1.94	2.21
Interactive module	Point-based feedback	+10	P3	0.12	0.93
			N2	0.74	1.63
		+1	P3	−0.27	1.00
			N2	0.34	0.82
		−5	P3	0.54	1.56
			N2	−1.74	2.43
		−10	P3	0.95	1.44
			N2	−2.55	2.49
	Type-based feedback	Positive (+10)	P3	0.12	0.93
			N2	0.74	1.63
		Neutral (+1)	P3	−0.27	1.00
			N2	0.34	0.82
		Negative (−5, −10)	P3	0.75	1.33
			N2	−2.14	0.81
	Punishment	Weak (−5) – Positive (+10)	FRN	−2.50	1.94
		Strong (−10) – Positive (+10)	FRN	−3.15	2.25

*The reported values are calculated mean amplitudes for P3 (300–700 ms) and N2 (200–300 ms)*.

*The FRN was calculated as a difference between the mean amplitudes for negative feedback and positive feedback*.

#### 3.1.1. P3

For P3 mean amplitude, which was measured between 300 and 700 ms, a 3 × 2 ANOVA comparing vertical electrode clusters [L, C, R] to trial type [Frequent, Infrequent] yielded no significant main effects of channel type [*F*_(2, 32)_ = 0.99, *p*_*c*_ = 0.37], significant main effects of trial type [*F*_(1, 16)_ = 11.72, *p* < 0.01], and no significant interactions between channel type and trial type [*F*_(2, 32)_ = 3.48, *p*_*c*_ = 0.055]. A 3 × 2 ANOVA on mean amplitude comparing horizontal electrode clusters [F, M, B] to trial type [Frequent, Infrequent] found no significant main effects of channel type *F*_(2, 32)_ = 2.88, significant main effects of trial type [*F*_(1, 16)_ = 11.17, *p* < 0.01], p_*c*_ = 0.10), and no significant interactions between channel type and trial type [*F*_(2, 32)_ = 0.21, *p*_*c*_ = 0.69].

#### 3.1.2. N2

For N2 mean amplitude, which was measured between 200 and 300 ms, a 3 × 2 ANOVA comparing [L, C, R] to [Frequent, Infrequent] yielded significant main effects of channel type [*F*_(2, 32)_ = 9.72, *p*_*c*_ < 0.01], significant main effects of trial type [*F*_(1, 16)_ = 77.12, *p* < 0.001], and significant interactions between trial type and channel type [*F*_(2, 32)_ = 6.47, *p*_*c*_ < 0.01]. Channel pairs [L, C] and [C, R] were significant (*p* < 0.01) but not [L, R] (*p* = 0.12). The interaction corrected paired *t*-test revealed no significant differences in any pair for frequent trials (*p* > 0.05) and yielded significant differences for [L, C] and [C, R] (*p* < 0.05) but not [L, R] (*p* = 1.00) for infrequent trials. A 3 × 2 ANOVA on mean amplitude comparing [F, M, B] to [Frequent, Infrequent] found significant main effects of channel type [*F*_(2, 32)_ = 11.39, *p*_*c*_ < 0.01], significant main effects of trial type [*F*_(1, 16)_ = 85.67, *p* < 0.001], and significant interactions between trial type and channel type [*F*_(2, 32)_ = 5.27, *p*_*c*_ < 0.05]. Channel pairs [F, B] and [M, B] were significant (*p* < 0.01) but not [F, M] (*p* = 1.00). Frequent trials yielded no significant differences between any pair of electrode clusters (*p* > 0.06) and infrequent trials yielded significant differences in [M, B] (*p* < 0.01) but not between any other pair (*p* > 0.08).

### 3.2. Interactive Module

**Figure 6:**
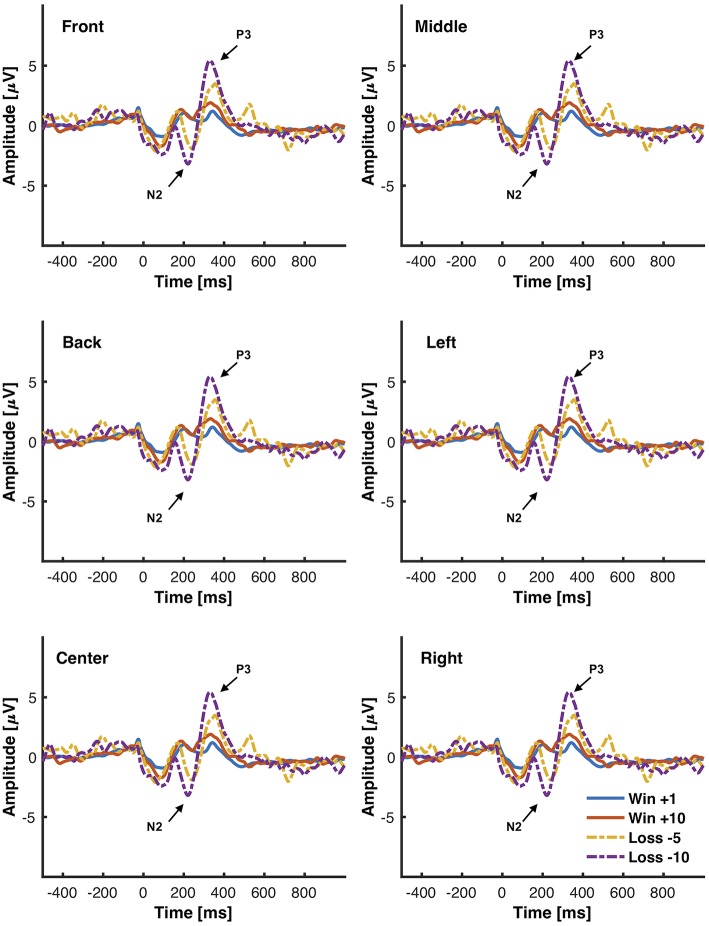
Grand average ERPs elicited from the interactive module in response to different magnitudes of positive (+10), negative feedback (−5, −10), and neutral feedback (+1). 500 ms of pre-stimulus and 1,000 ms of post-stimulus activity is shown. Positive voltage is plotted up. Vertical bars indicate feedback stimulus onset.

The objective for this study was to analyze the P3, N2, and FRN components using the chosen low-cost wearable EEG system in an interactive environment that provided feedback. ERPs for electrode clusters are plotted in Figure 6. ERP confidence intervals are plotted in Figures 5B,C. The mean amplitudes for P3 and N2 are reported in Table 1. The power spectral density is reported in Figure 4.

#### 3.2.1. Point-Based Feedback: +10, +1, −5, −10

##### 3.2.1.1. P3

In the interactive module, a 3 × 4 ANOVA on P3 mean peak amplitude between 300 and 700 ms comparing [L, C, R] to [+10, +1, −5, −10] found significant main effects of channel type [*F*_(2, 30)_ = 5.79, *p*_*c*_ < 0.05], significant main effects of trial type [*F*_(3, 45)_ = 8.78, *p*_*c*_ < 0.01], and no significant interactions between trial type and channel type [*F*_(6, 90)_ = 1.90, *p*_*c*_ = 0.14]. Channel pairs [L, C] and [L, R] were significant (*p* < 0.05) but [C, R] was not (*p* = 0.15). Most trial pairs were significant (*p* < 0.01) except [+10, −5] and [−5, −10] (*p* > 0.11). A 3 × 4 ANOVA on peak mean amplitude comparing [F, M, B] to [+10, +1, −5, −10] found significant main effects of channel type [*F*_(2, 30)_ = 5.27, *p*_*c*_ < 0.05], significant main effects of trial type [*F*_(3, 45)_ = 8.96, *p*_*c*_ < 0.01], and no significant interactions between trial type and channel type [*F*_(6, 90)_ = 1.83, *p*_*c*_ = 0.14]. Channel pairs [F, B] and [F, M] were significant (*p* < 0.05) but [M, B] was not (*p* = 0.21). Most trial pairs were significant (*p* < 0.01) except [+10, −5] and [−5, −10] (*p* > 0.89).

##### 3.2.1.2. N2

A 3 × 4 ANOVA on N2 mean amplitudes measured between 200 ms and 300 ms comparing [L, C, R] to [+10, +1, −5, −10] found no significant main effects of channel type [*F*_(2, 30)_ = 2.01, *p* = 0.15), significant main effects of trial type [*F*_(3, 45)_ = 20.69, *p*_*c*_ < 0.001], and no significant interactions between trial type and channel type [*F*_(6, 90)_ = 0.95, *p*_*c*_ = 0.42]. Trial pair [+10, +1] was not significant (*p* = 1.00) but all other pairs were significant (*p* < 0.05). A 3 × 4 ANOVA on mean amplitudes comparing [F, M, B] to [+10, +1, −5, −10] found significant main effects of channel type [*F*_(2, 30)_ = 3.35, *p* < 0.05], significant main effects of trial type [*F*_(3, 45)_ = 25.92, *p*_*c*_ < 0.001], and significant interactions between trial type and channel type [*F*_(6, 90)_ = 2.78, *p* < 0.05]. Channel pair [F, M] was not significant (*p* = 1.00) but all other pairs were (*p* < 0.05). Trial pair [+10, +1] was not significant (*p* = 0.58) but all other pairs were significant (*p* < 0.05). Within bins, there were no significant difference between channels (*p* > 0.27). Within channels, trial pairs [+10, −5], [+10, −10], and [+1, −10] were significant (*p* < 0.05) and all other pairs were not significant (*p* > 0.12).

#### 3.2.2. Type of Feedback: Positive, Neutral, and Negative

The FRN is traditionally calculated as either an analysis between N2 components elicited from different feedback or as a difference wave between negative and positive feedback. This section analyzed feedback as elicited from an interactive module that allowed participants freedom to make decisions. The data was separated into high positive feedback (+10) and negative feedback (−5 and −10). The mean amplitudes and standard deviations for P3 and N2 are reported in Table 1. The 95% confidence interval grand average ERP between all electrodes for high positive, negative, and neutral feedback is shown in Figure 5B. The 95% confidence interval grand average of the FRN which was determined by the difference between negative and high positive feedback is shown in Figure 5C.

A 3 × 3 ANOVA on P3 amplitudes comparing [L, C, R] to [Positive, Neutral, Error] significant main effects of channel type [*F*_(2, 32)_ = 5.15, *p*_*c*_ < 0.05], found significant main effects of trial type [*F*_(2, 32)_ = 14.54, *p*_*c*_ < 0.001], and no significant interactions between trial type and channel type [*F*_(4, 64)_ = 0.93, *p*_*c*_ = 0.40]. Channel pairs [L, C] and [L, R] were significant (*p* < 0.001) and [C, R] was not. All trial pairs were significant (*p* < 0.001). A 3 × 3 ANOVA on P3 amplitudes comparing [F, M, B] to [Positive, Neutral, Error] found significant main effects of channel type [*F*_(2, 32)_ = 4.37, significant main effects of trial type [*F*_(2, 32)_ = 16.70, *p*_*c*_ < 0.001], p_*c*_ < 0.05], and no significant interactions between trial type and channel type [*F*_(4, 64)_ = 2.23, *p*_*c*_ = 0.11]. Channel pair [F, B] was significant (*p* < 0.001) and [M, B] and [F, M] were not (*p* > 0.084). All trial pairs were significant (*p* < 0.001).

A 3 × 3 ANOVA on N2 amplitudes comparing [L, C, R] to [Positive, Neutral, Error] found no significant main effects of channel type [*F*_(2, 32)_ = 2.49, significant main effects of trial type [*F*_(2, 32)_ = 26.45, *p*_*c*_ < 0.001], *p*_*c*_ = 0.11], and no significant interactions between trial type and channel type [*F*_(4, 64)_ = 1.40, *p*_*c*_ = 0.257]. Trial pairs [Positive, Error] and [Neutral, Error] were significant (*p* < 0.001) but [Positive, Neutral] was not (*p* = 0.28). A 3 × 3 ANOVA on N2 amplitudes comparing [F, M, B] to [Positive, Neutral, Error] found significant main effects of channel type [*F*_(2, 32)_ = 6.54, *p*_*c*_ < 0.01], significant main effects of trial type [*F*_(2, 32)_ = 34.88, *p* < 0.001], and no significant interactions between trial type and channel type [*F*_(6, 64)_ = 2.72, *p*_*c*_ = 0.05]. [F, B] and [B, M] were significant (*p* < 0.01) but [F, M] was not (*p* = 1).

#### 3.2.3. FRN: Weak and Strong Negative Feedback

This section analyzed FRNs from different levels of negative feedback as elicited from an interactive module that allowed participants freedom to make decisions. The data was separated into weak negative feedback (−5) and strong negative feedback (−10) and subtracted by high positive feedback (+10). The mean amplitudes and standard deviation for the FRN are reported in Table 1.

A 3 × 2 ANOVA on FRN mean amplitudes comparing [L, C, R] to FRN type [Low, High] found, no significant main effects of channel type [*F*_(2, 32)_ = 0.41, *p*_*c*_ = 0.63], no significant main effects of FRN type [*F*_(1, 16)_ = 2.41, *p* = 0.14] and no significant interactions between trial type and channel type [*F*_(2, 32)_ = 1.56, *p*_*c*_ = 0.21]. A 3 × 2 ANOVA on FRN mean amplitudes comparing [F, M, B] to [Low, High] found no significant main effects of trial type [*F*_(1, 16)_ = 2.05, *p* = 0.17], significant main effects of channel type [*F*_(2, 32)_ = 4.10, *p*_*c*_ < 0.05], and significant interactions between FRN type and channel type [*F*_(2, 32)_ = 4.03, *p*_*c*_ < 0.05]. [F, M] and [B, M] were significant (*p* < 0.05) and [F, B] was not (*p* = 1.00). Within low, channel pair [M, B] was significant (*p* < 0.05) but all others were not (*p* > 0.52). Within high, no channel pairs were significant (*p* = 1.00).

## 4. Discussion

The study had two main objectives: (1) Analyze ERP components from a low-cost wearable EEG system in a standard oddball paradigm, (2) Analyze the P3, N2, and FRN components using a low-cost wearable EEG system in an interactive environment. The first hypothesis was that the low-cost wearable EEG would detect statistically significant P3 components for infrequent stimuli in a standard oddball paradigm. The second hypothesis was that the low-cost EEG system can detect statistically significant ERP components elicited in response to feedback in an interactive module. The follow-up hypotheses for the second objective was that, when comparing negative and positive feedback, negative feedback would elicit a larger N2 component and, when comparing weak and strong negative feedback, stronger negative feedback would elicit a larger FRN component. The grand average of the power spectral density (PSD), as shown in Figure 4, shows a small peak located around 10 Hz in both the oddball task and the interactive module. The results indicate that the OpenBCI low-cost EEG system is capable of detecting differences in ERP components in the oddball paradigm and differences in components as measured from positive and negative feedback in an interactive module. This could potentially aid in the development of neurometrics for consumers using a low-cost affordable EEG product who are interested in feedback monitoring.

With the visual oddball paradigm, we verified that the OpenBCI can collect ERPs with statistically significant differences between P3 and N2 amplitude between frequent and infrequent stimuli as shown in section 3.1 and Figure 5A which is consistent with the first hypothesis. These statistical results are in line with reported results from prior research that infrequent stimuli elicit larger and more prominent P3 and N2 peaks (Treder and Blankertz, 2010; Debener et al., 2012, 2015; Badcock et al., 2013; Halder et al., 2013; De Vos et al., 2014; Frey, 2016; Vourvopoulos and Badia, 2016; Weschke and Niedeggen, 2016; Krigolson et al., 2017; Mathewson et al., 2017; Ratti et al., 2017; Dehais et al., 2019; Kam et al., 2019). When we directly compare the amplitudes as calculated to those of prior research as shown in Table 2, the infrequent P3 and N2 mean amplitudes within this work are within one standard deviation of the distribution of the same results as extracted from the literature as shown in Table 4. However, the P3 and N2 amplitudes as elicited by the frequent stimulus from the OpenBCI were more prominent. This discrepancy could be explained by the differences of calculating the amplitude. The literature found reported the maximum amplitude within a time range whereas this current study reports the mean amplitude within the same time range.

**Table 2:** P3 and N2 amplitudes from oddball task literature.

	**Amplifier**		**Electrode**		**P3 (**μV**)**		**N2 (**μV**)**	
**Study**	**Name**	**Type**	**Num**	**Type**	**Infreq**	**Freq**	**Infreq**	**Freq**
Treder and Blankertz (2010)^*^	actiCAP	Clinical	64	Wet	2.60	0.50	−3.25	−0.50
Halder et al. (2013)^*^	BrainAmp	Clinical	63	Wet	4.99	−1.50	−3.25	−2.50
Badcock et al. (2013)	Neuroscan 4.3	Clinical	16	Wet	3.55	0.30	−0.15	−0.78
Frey (2016)^*^	g.USBamp	Clinical	16	Wet	1.65	^**^	−0.40	^**^
Weschke and Niedeggen (2016)	BioAmplifiers	Clinical	3	Wet	5.52	0.56	−0.51	−0.66
Krigolson et al. (2017)^*^	actiCAP	Clinical	64	Wet	12.50	2.67	4.87	2.67
Mathewson et al. (2017)	actiCAP	Clinical	15	Wet	4.00	0.53	^**^	^**^
Kam et al. (2019)^*^	ActiveTwo	Clinical	64	Wet	1.50	−0.53	−1.00	0
Mathewson et al. (2017)	actiCAP	Clinical	15	Dry	3.00	1.10	^**^	^**^
Kam et al. (2019)^*^	F1 1.0 EEG	Clinical	33	Dry	1.46	−0.33	−2.00	0.10
Debener et al. (2012)	Emotiv Epoc	Low-cost	16	Wet	10.00	−0.20	^**^	^**^
Badcock et al. (2013)^*^	Emotiv Epoc	Low-cost	16	Wet	3.94	0.55	−0.15	−0.65
Debener et al. (2015)^*^	EEGrid	Low-cost	16	Wet	3.50	0.50	−1.00	−0.75
Frey (2016)^*^	OpenBCI	Low-cost	16	Wet	1.50	^**^	−0.25	^**^
Dehais et al. (2019)	Enobio	Low-cost	6	Dry	3.51	−0.50	^**^	^**^
**Results**	**OpenBCI**	**low-cost**	**16**	**Dry**	**4.33**	**2.48**	**−2.63**	**1.94**

* *These studies reported some of the amplitudes through figures. These amplitudes are estimated at peak*.

** *Amplitudes not reported*.

*The bolded values are the results collected from this specific study. They were bolded to emphasize the comparison between contemporary studies*.

The interactive module was not only able to elicit ERPs in response to feedback, but also elicit statistically significant ERPs between different point values of feedback (+10, +1, −5, −10) and different types of feedback (positive, negative, neutral) which is consistent with the second hypothesis and the first of the follow-up hypotheses as shown in section 3.2 and Figure 5B. However, the results indicated that there were no significant differences between FRN types (weak vs. strong negative feedback) and that there was one significant interaction between FRN type and channel group when comparing the Front, Middle, and Back electrodes. This was opposite of expectation as reward expectation and magnitude has been seen to influence the P3 and N2 component (Sato et al., 2005; Pornpattananangkul and Nusslock, 2015; Philiastides et al., 2018). We were able to show that a larger N2 component would be elicited when the participant encountered something that was contrary to expectation of the solution construct. The results related to the N2 component are consistent with literature (Cohen et al., 2007; Potts, 2011; Gheza et al., 2018; Krigolson, 2018; Yi et al., 2018; Somon et al., 2019; Tunison et al., 2019). However, when we compared the calculated mean amplitudes to existent literature as shown in Table 3, the calculated amplitudes were smaller. However, the calculated FRN from this study was the within the same range as the FRN from existent literature as shown in Table 4. Again, similar to the oddball task, the discrepancies might be explained by the differences in calculating the amplitudes of the peaks. However, as the FRN is calculated from a difference between amplitudes taken from two types of feedback, there is little discrepancy between the FRN amplitude. We hypothesized that the FRN would be distinguishable strictly between different levels of negative feedback although the results indicated the opposite as shown in Table 1. This result could be from the nature of the interactive module as it is far more likely to achieve positive feedback than either of the negative feedbacks which has been shown to influence the FRN (Walsh and Anderson, 2012; Warren and Holroyd, 2012; Gheza et al., 2018; Glazer et al., 2018; Krigolson, 2018; Somon et al., 2019). In a future study, it might be worth investigating the effect of increasing the probability of negative feedback as perceived by the subjects.

**Table 3:** P3, N2, and FRN amplitudes from feedback-related event related potential literature.

	**Amplifier**		**Electrode**		**P3 (**μV**)**		**N2 (**μV**)**		**FRN (μV)**
**Study**	**Name**	**Type**	**Num**	**Type**	**Reward**	**Punish**	**Reward**	**Punish**	**Diff**
Potts (2011)^*^	Geodesic 250	Clinical	128	Wet	0.25	−0.25	0.75	4.50	−3.17
Krigolson et al. (2017)^*^	actiCAP	Clinical	64	Wet	11.50	10.50	7.50	1.50	−5.67
Pornpattananangkul and Nusslock (2015)	Neuroscan	Clinical	24	Wet	18.00	12.00	−2.25	−3.50	−0.17
Gheza et al. (2018)	ActiveTwo	Clinical	64	Wet	3.10	3.20	2.42	−0.41	−2.83
Somon et al. (2019)^*^	actiCAP	Clinical	75	Wet	5.00	10.00	7.50	4.50	−3.17
**Results**	**OpenBCI**	**Low-cost**	**16**	**dry**	**0.12**	**0.75**	**0.74**	**−2.14**	**−3.15**

* *These studies reported some of the amplitudes through figures. These amplitudes are estimated at peak*.

*The bolded values are the results collected from this specific study. They were bolded to emphasize the comparison between contemporary studies*.

**Table 4:** Mean comparison between literature and results.

**Task**	**Stimulus**	**Peak**	**Literature (μV)**	**Results (μV)**
Oddball	Infrequent	P3	4.22 (3.15)	4.33 (4.12)
		N2	−0.64 (2.16)	−2.63 (2.15)
	Frequent	P3	0.28 (0.99)	2.48 (2.74)
		N2	−0.34 (1.35)	1.94 (2.21)
Interactive module (Feedback related ERPs)	Reward	P3	7.57 (7.15)	0.12 (0.93)
		N2	3.18 (4.28)	0.74 (1.64)
	Punish	P3	7.09 (5.32)	0.75 (1.33)
		N2	0.52 (2.91)	−2.14 (0.81)
	Punish—Reward	FRN	−2.42 (2.29)	−3.15 (2.25)^*^

* *The interactive module results for strong punishment FRN*.

This current study reviewed time-related ERP features in relation to an oddball task and an interactive task through the perspective of a low-cost EEG system. However, there are several limitations to this study and, as a result, multiple future directions that could be investigated. Several challenges relate to the hardware of the EEG system. The largest challenge of using this particular arrangement of wearable low-cost EEG system was data quality. Incorporating dry electrodes into a low-cost system requires adequate placement and decent contact with the scalp to collect sufficiently high quality data. The stretch headcap used in these experiments aided in adding sufficient contact. However, participants indicated discomfort when wearing the assembly with dry electrodes for long periods of time. In addition, for participants with small head sizes or large amounts of hair, sufficient contact was difficult to achieve which led to a reduction in data quality. Because of the loss in data quality, electrodes were averaged together in the analysis groups (Front, Middle, Back, Left, Center, Right). As the OpenBCI system is compatible with wet electrodes, future studies that are concerned with data quality and wish to use OpenBCI systems should proceed with traditional electrodes. In the next iteration of research, the data quality from this paper could be further improved by using the Riemannian Artifact Subspace Reconstruction (Blum et al., 2019; Dehais et al., 2019). However, even with these challenges, we were able to quantify differences in the N2 and P3 components between different stimuli and types of feedback. This study also only analyzes one low-cost EEG system with flexible electrode positioning. There are no recommendations from this study for the treatments of EEG systems that employ inflexible electrode positioning other than to ensure good contact between electrode and scalp (Krigolson et al., 2017; Ratti et al., 2017).

It was also made apparent through prior testing and through literature review that the EEG system would lag in delivering data to the computer (Debener et al., 2012; Frey, 2016; Vourvopoulos and Badia, 2016; Krigolson et al., 2017; Ratti et al., 2017). In order to measure this lag, we developed a circuit with a photoresistor that sent digital indicator to an auxiliary port on the onset of a stimulus. The average delay between the marked stimulus onset time for Psychopy and the OpenBCI marked time was 47.23 ms with a standard deviation of 3.56 ms, which was remarkably consistent. The photoresistor port can serve as a safeguard or as a calibration method to measure delay of stimulus onset. As long as the computer program recording user responses marks time based on stimulus onset, the user should be confident in the timing reliability of the OpenBCI. However, for all consumer-grade EEG system, EEG time is marked by the receiving computer. The OpenBCI system required some reprogramming to transmit the time as measured by the system's arduino. The time was therefore measured relative to data submission to the computer rather than data received by the computer. All of these were modifications made outside of unboxing the OpenBCI system. In working with presently available consumer product EEG systems to study ERPs, users still need to take these limitations into account or implement system modifications as was done in the present study. In addition, the study was also only limited to analyzing ERP features after standard time data collection. However, it is possible to analyze these ERP features while live streaming (Abujelala et al., 2016; Agapov et al., 2016). The planned future work has already incorporated Python-based EEG analysis to investigate single-trial classifications and will forgo independent component analysis to remove eye blinks in favor of incorporating electrooculography (EOG) to remove those specific artifacts. Other than investigating the power spectral density as shown in Figure 4, the current study was strictly limited to investigating the time-related features of the ERP responses. However, other studies, especially those regarding feedback related event related potentials, have investigated the frequency band activity through traditional systems and found that reward-related feedback increases the beta power over the right-frontal area and theta power increases with punishment (Cohen et al., 2007; HajiHosseini and Holroyd, 2015; Glazer et al., 2018; Masaki et al., 2018; Philiastides et al., 2018). In addition, low-cost wireless EEGs have been shown to be capable of accurately measuring band power, even in active circumstances (Ratti et al., 2017; Dehais et al., 2019). The future direction of the study would investigate frequency band activity related to the ERP as elicited by the interactive module.

A low-cost EEG, with all of its limitations, may not be able to capture all the minute distinctions between different types of errors as shown from the interactive module results. However, in general, the study shows that the low-cost EEG has the distinct advantage of providing consumers the affordable ability to measure ERP components related to feedback monitoring in less structured computer environments. With improved modifications to the wearable EEG systems, such as a synchronization circuit and flexible electrode position as shown in section 2.3.2, consumers can reliably collect these components in these environments and, potentially, monitor their own performance over time using these identified ERP components as neuro-based metrics and relying on future research into real-time processing. By extension, researchers can use both clinical and low-cost EEG systems to collect ERP components in context of less structured and interactive computer environments and potentially expand ERP studies beyond classic scenarios.

## 5. Conclusion

In this study, we examined the P3, N2, and FRN components in an ERP elicited in response to stimuli from a traditional oddball paradigm and provided feedback in an interactive module that encouraged learning. We were able to accomplish significant results from a low-cost wearable EEG device. Results from the oddball paradigm determined that, with slight modifications, an OpenBCI can collect statistically significant ERPs in ERP studies. The results from the interactive module indicated distinctions between the ERPs elicited from neutral feedback, high reward feedback, and negative feedback. In studies conducted by different EEG devices, the P3 and N2 components were shown to be comparable in the oddball paradigms and the FRN components were shown to be comparable to similar FRN studies as shown in Table 4. The research indicates a low-cost portable system is capable of detecting statistically significant ERPs in response to both positive and negative feedback even in interactive settings. Our method utilized a system that costs <$1,500 dollars that required few modifications. The results from this study imply that consumers can use this specific affordable wearable low-cost EEG to measure ERP components related to feedback monitoring in less structured computer environments.

## Data Availability

The datasets generated for this study are available on request to the corresponding author.

## Ethics Statement

This study was carried out in accordance with the recommendations of U.S. Department of Health and Human Services federal regulation 45 C.F.R. 46, Dartmouth College Institutional Review Board (IRB) with written informed consent from all subjects. All subjects gave written informed consent in accordance with the Declaration of Helsinki. The protocol was approved by the Dartmouth IRB.

## Author Contributions

JQ, MC, and SD designed the research. JQ performed the experiment and analyzed the data. JQ and SD drafted the work and revised the manuscript.

### Conflict of Interest Statement

The authors declare that the research was conducted in the absence of any commercial or financial relationships that could be construed as a potential conflict of interest.
