# Supplementary figures and images for: Assessing Feedback Response With a Wearable Electroencephalography System
Source: Front Hum Neurosci. 2019 Jul 25;13:258. doi: 10.3389/fnhum.2019.00258 (PMC6669939; doi:10.3389/fnhum.2019.00258)

## Supplementary Material

### 0.1 Figures

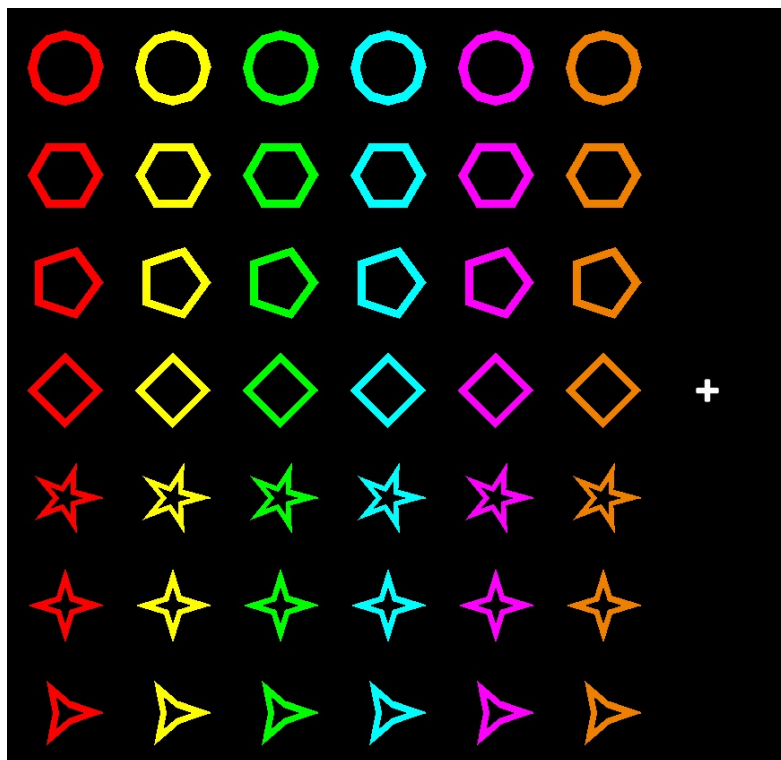

**Figure S1.** Library of Stimuli

Supplement: Supplementary file 1 [file Data_Sheet_1.PDF]
